# Implantation of Islets Co-Seeded with Tregs in a Novel Biomaterial Reverses Diabetes in the NOD Mouse Model

**DOI:** 10.1007/s13770-024-00685-7

**Published:** 2024-12-30

**Authors:** Diana M. Elizondo, Lais L. de Oliveira Rekowsky, Ayane de Sa Resende, Jonathan Seenarine, Ricardo Luis Louzada da Silva, Jamel Ali, Dazhi Yang, Tatiana de Moura, Michael W. Lipscomb

**Affiliations:** 1 Department of Pharmacology, University of Minnesota, Minneapolis, MN 55455 USA; 2 Center for Immunology, University of Minnesota, Minneapolis, MN 55455 USA; 3 Department of Biological Sciences, University of Maryland Baltimore County, Baltimore, MD 21250 USA; 4 Department of Morphology, Federal University of Sergipe, São Cristóvão, Brazil; 5 Department of Chemical and Biomedical Engineering, FAMU‐FSU College of Engineering, Tallahassee, FL 32310 USA; 6 Acrogenic Technologies Inc., Rockville, MD 20850 USA

**Keywords:** NOD, Tregs, Biomaterial

## Abstract

**Background:**

Type 1 diabetes (T1D) results in autoreactive T cells chronically destroying pancreatic islets. This often results in irreplaceable loss of insulin-producing beta cells. To reverse course, a combinatorial strategy of employing glucose-responsive insulin restoration coupled with inhibiting autoreactive immune responses is required.

**Methods:**

Non-obese diabetic mice received a single intraperitoneal implantation of a novel biomaterial co-seeded with insulin-producing islets and T regulatory cells (Tregs). Controls included biomaterial seeded solely with islets, or biomaterial only groups. Mice were interrogated for changes in inflammation and diabetes progression via blood glucose monitoring, multiplex serum cytokine profiling, flow cytometry and immunohistochemistry assessments.

**Results:**

Islet and Tregs co-seeded biomaterial recipients had increased longevity, insulin secretion, and normoglycemia through 180 days post-implantation compared to controls. Serum profile revealed reduced TNFα, IFNγ, IL-1β and increased IL-10, insulin, C-Peptide, PP and PPY in recipients receiving co-seeded biomaterial. Evaluation of the resected co-seeded biomaterial revealed reduced infiltrating autoreactive CD8 + and CD4 + T cells concomitant with sustained presence of Foxp3 + Tregs; further analysis revealed that the few infiltrated resident effector CD4^+^ or CD8^+^ T cells were anergic, as measured by low levels of IFNγ and Granzyme-B upon stimulation when compared to controls. Interestingly, studies also revealed increased Tregs in the pancreas. However, there was no restoration of the pancreas beta cell compartment, suggesting normoglycemia and production of insulin levels were largely supported by the implanted co-seeded biomaterial.

**Conclusion:**

These studies show the efficacy of a combinatorial approach seeding Tregs with pancreatic islets in a novel self-assembling organoid for reversing T1D.

## Introduction

Type 1 diabetes (T1D) is a chronic and debilitating disease marked by immune cell infiltration and destruction of insulin producing-beta cells within the pancreas, with autoreactive CD8 + T cells specifically recognizing and targeting these cells for destruction [1–3]. In tandem, these CD8 + T cells receive *help* in promoting autoreactive effector functions from infiltration of CD4 + T cells [4–7]. Collectively, these autoimmune responses destroy the beta cell compartment leading to impaired insulin production, which results in elevating blood glucose levels towards hyperglycemic states. Thus, significant challenges impeding successful outcomes in beta cell replacement strategies continue to be infiltration of the host’s autoreactive T cells into the newly grafted tissues [8,9].

Insulin restoration approaches utilizing syngeneic or donor-derived pancreatic islets have been moderately successful. Minimal invasive approaches have further increased wide-use of beta cell replacement strategies. However, all current islet transplantation approaches require immunosuppression regimens such as neutralizing antibodies to CD3 and/or TCR to suppress allo- and/or autoreactive T cells from rejecting grafted tissues [10,11]. Unfortunately, use of immunosuppressive agents abrogates natural host immunity and can lead to chronic infections and increased morbidities.

Approaches to encapsulate beta cells as microcarriers have been met with moderate success [8,12–15]. As opposed to transplant of free islets or insertion into the kidney capsule, the aim of encapsulation or use of a bioscaffold is to protect the cells from the host immune system by an artificial membrane. Several strategies have shown a potential of success via subcutaneous (subq) implantation, as opposed to intraperitoneal, which represents an attractive minimal invasive procedure [16,17]. Unfortunately, no system has adequately provided long-term evasion from circulating myeloid cells or autoreactive T cells [16,18–21]. As such, reversal of diabetes is short lived.

Importantly, the body produces Foxp3 + CD4 + T cells (Tregs) as a natural counterbalance to restrain excessive immunity [22–25]. Low levels of circulating and pancreas-resident Tregs is associated with type 1 diabetes onset and progression [26]. Interestingly, both monoclonal beta cell antigen-specific and polyclonal Tregs have shown a strong ability to effectively suppress autoreactive T cells in T1D [27–30]. Therefore, employing Tregs to create a suppressive microenvironment within the pancreas islet graft would support long-term survivability by suppressing infiltrating autoreactive T cells [30–33].

Prior work by our laboratory group employed a novel bioscaffold seeded with islets to reverse hyperglycemia in the non-obese diabetic (NOD) and the streptozotocin-induced diabetic mouse models. Implantation of this islet-seeded biomaterial resulted in organoid formation coupled with vasculogenesis in both diabetic recipient models [8]. This resulted in successful restoration of insulin, re-achievement of normoglycemia and healthy metabolic indicators. However, within the NOD model, which is driven by autoimmunity, the studies observed failure of the organoid several weeks post-implantation. Investigations revealed the autoreactive T cells that targeted the pancreas islets were now infiltrating into the newly seeded biomaterial organoid.

Therefore, to sustain effective reversal of hyperglycemic states, a combinatorial approach of an islet-seeded organoid in conjunction with a method to effectively suppress autoimmunity within the immediate microenvironment is needed. To directly address, the studies herein combined seeding of syngeneic pancreatic islet with CD4 + Foxp3 + Tregs for adoptive transfer into recipient NOD mice. The premise is that the seeded Tregs would take residence within the organoid microcarrier to restrain autoimmune insults to allow long-term viability of the glucose-responsive insulin-producing beta cells to achieve normoglycemia and reverse diabetes.

## Materials and methods

### Biomaterial assembly

For biomaterial preparation, the patented approach includes selectively oxidizing cellulose, covalently cross-linking 2,3 di-aldehyde cellulose with polyamine polymers and reducing the carbon–nitrogen double bonds of the imines. Importantly, 2,3 di-aldehyde cellulose covalently cross-links with the functional block polymers to form the polyamine cellulosic copolymers with a three-dimensional densely interlocked network. Amine groups were protonated under an aqueous environment with a pH lower than 9 allowing the positively charged copolymeric scaffold to form hydrogel matrices. Functional group characterization of the material was evaluated using Fourier Transform Infrared Spectroscopy (FTIR) analysis. Complete details of the patented biomaterial is available through patent US 20180094080.

### SEM sample preparation and analysis

Aggregated pieces of islet-seeded biomaterial were transferred into 2% buffered glutaraldehyde. Samples were fixed overnight at 4 °C. After fixation, samples were rinsed with 0.1 M HEPES buffer 3 × for 5 min each with gentle agitation followed by extensive rinsing. Preparations were dehydrated using an alcohol serial dehydration approach. Next, samples were chemically dried through incubation in gradients of HMDS for 15 min each prior to placing onto clean silicon chips and sputter coated with a thin film (~ 15 nm) of palladium. Ultrastructure images were acquired via FEI Helios G4 field emission scanning electron microscope operated at an acceleration voltage of 3 kV.

### Animals

Female non-obese diabetic (NOD; ShiJL/T) mice served as recipients. Both male and female NOD mice containing green fluorescent protein (GFP) expressed under the Foxp3 promoter (NOD-Foxp3^gfp^) were utilized to generate Foxp3^+^ T regulatory (Treg) cells. Wild type (C57BL/6; WT) and NOD.SCID mice were used for controls. All animals were purchased from The Jackson Laboratory and housed at University of Minnesota and Howard University under the IACUC protocols 2203-39881A, GSAS1201 and GSAS1501.

### Glucose monitoring

Glucose monitoring using the Aviva Accu-Check (Roche; Indianapolis, IN, USA) glucometer by tail vein prick was performed twice a week to assess onset and progression of diabetes by measuring glucose levels in both STZ-induced and NOD mice. Tails of mice were pricked to collect 2 µl of blood for immediate reading on glucometer test strips. Diabetes was identified by two consecutive values > 250 mg/dL of non-fasted mice. Pre-diabetic states identified as transient values 150–300 mg/dL [34,35]. Normoglycemia was measured at levels of < 150 mg/dL.

### Isolation of syngeneic pancreatic islets

NOD mice were dissected using a 40X wide-field stereomicroscope for pancreas islet isolation. Islet purification steps were performed using a modified protocol from Stull et al. [36]. Briefly, pancreas was inflated with 200 µl of collagenase type IV at a concentration of 0.5 mg/mL in HBSS prior to injection through the hepatic vein using a 27-gauge needle. The pancreas was digested with 2 mg/mL collagenase type IV (Thermo Fisher Scientific; Waltham, MA, USA) for 30 min at 37 °C. Next, suspension was mixed gently prior to centrifugation for 1 min. The pellet was resuspended in HBSS and overlaid with pre-mixed histopaque 1100 and histopaque 1077 (Sigma Aldrich; St. Louis, MO, USA) prior to centrifugation for 20 min at 330xg. The middle white layer containing islets was carefully collected and transferred to a new tube containing RPMI media. In some studies, CD31 + and VEGFR + endothelial cells were depleted using magnetic beads; primary antibodies purchased from BioLegend (BioLegend, San Diego CA, USA) and secondary antibodies conjugated to magnetic microbeads from Qiagen (Germantown, MD, USA). Islets were allowed to recover by incubating at 37 °C for 24 h. Next, ~ 2000 islets were hand-picked using a stereomicroscope and seeded into the biomaterial at indicated islet-to-cell ratio to be employed.

### Generation and isolation of tregs

Using a variation of the approach by Tang Q et al. [29], lymph nodes (cervical, brachial, axillary, and inguinal) were harvested from NOD-Foxp3^gfp^ male and female mice and CD4 + CD25- T cells were isolated using CD4 and CD25 magnetic beads (Miltenyi Biotec; Gaithersburg, MD, USA). In some studies, naive CD4 + CD25-Foxp3^gfp^ cells were sorted by FACS to isolate the pure population of precursors. The FACS- or magnetic bead-sorted naive CD4 + T cells were then *in vitro *cultured with TGFβ (5 ng/mL) and IL-2 (20 IU/mL) cytokines and neutralizing antibodies to IL-12 (10 ng/mL), IL-4 (10 ng/mL) and IFNγ (20 ng/mL) (BioLegend) for 14 days in the presence of CD3/CD28 Dynabeads (Thermo Fisher Scientific). FACS-sorting was then used to purify the expanded CD4 + CD25 + GFP + Tregs for co-seeding in the biomaterial.

### Cell seeding in biomaterial and intraperitoneal injection

Biomaterial was disinfected in 70% ethanol and then washed extensively with deionized water. After washing, the biomaterial was cultured in RPMI overnight prior to beginning of studies. Next, biomaterial was cultured with isolated pancreatic islets and Foxp3^gfp^ Tregs in RPMI media supplemented with FBS and incubated at 37 °C. After two weeks of *in vitro* culture supplemented with 50 ng/mL of IL-7 and 25 ng/mL of IL-2, islet and Treg co-seeded biomaterial was sheared 5 times by passing through a 27-gauge needle 24 h prior to implantation in diabetic recipient mice. The amount of islet and Treg co-seeded biomaterial used for injection per mouse held approximately 500 islets and 10^4^ CD4 + Foxp3^gfp^ + Tregs. The sheared cell-seeded biomaterial was washed extensively in PBS to remove residual media and serum prior to re-suspension to 200 µl with PBS for intraperitoneal injection into diabetic recipient NOD mice. Injection of empty sheared biomaterial, islets-seeded biomaterial or Tregs intravenous or intraperitoneal injected served as additional comparative groups.

### Biomaterial cryosection and fluorescence microscopy

Excised Far-red labeled islet-seeded biomaterial from implanted mice were fixed in 3% PFA and further transferred into a 30% sucrose solution. Sections were prepared at 10 or 20 µm thickness using a standard automated cryosectioner (Cryostat NX70; Thermo Fisher). Preparations were transferred onto poly-L-lysine coated glass slides and permeabilized using 0.3% Triton-X solution, followed by blocking with 0.2% BSA in permeabilization buffer. Sections were stained with insulin CD31 (clone MEC 13.3) followed by extensive washing. Sections were co-stained with DAPI (Thermo Fisher) nuclear staining dye prior to mounting with cover slips. Slides were imaged using the Olympus FSX100 (Olympus, Waltham MA) or Evos FL (Thermo Fisher) fluorescence microscope.

### Dissociation of cells from the islet-seeded biomaterial

Resected pancreatic islet-seeded biomaterial from recipient mice was centrifuged down prior to resuspension in collagenase type IV (Thermo Fisher Scientific). The mixture was incubated on a rotating platform at 37 °C for 1 h. Next, cells were vortexed and strained using a 40 µm cell strainer. Dissociated cells were prepared for flow cytometry analyses.

### Flow cytometry

Cell surface staining was performed with PBS supplemented with 1 mM EDTA and 2.5% bovine serum (FACS buffer). Cells were washed with FACS buffer prior to extracellular staining with fluorochrome-tagged antibodies. Dilutions were antibody specific, but roughly 10 µl of a 10 µg/mL working concentration was utilized per 2 × 105 cells. Respective isotype controls were used in all assays. Cells were then fixed with 3% paraformaldehyde (PFA) in PBS. For intracellular antibody labeling, fixed cells were permeabilized with 0.2% saponin in PBS. Next, primary antibodies or isotype controls were added at approximately 10 µg/mL concentrations followed by washing and subsequent staining with secondary fluorochrome-labeled antibodies. Cells were acquired on a BD FACSVerse or Accuri C6 flow cytometric analyzer (BD Biosciences, San Jose CA, USA). Datasets were analyzed using FlowJo v10 (FlowJo LLC; Ashland OR, USA).

### Luminex multiplex and ELISA assays

Far Red dye (Thermo Fisher Scientific) was used to label islets prior to seeding in the biomaterial. The dye allows for visualization of islets in the biomaterial for several days *in vitro*. For multiplex analyses, blood was collected from mice weekly prior to and post-implantation of the cell-seeded biomaterial. Blood was processed into serum prior to adding to a mixture of color-coded beads pre-coated with analyte-specific capture antibodies. Biotinylated detection antibodies specific to the analytes of interest were added to form the antibody-antigen sandwich prior to reading on the Luminex MAGPIX Analyzer (Luminex; Austin TX, USA).

### Statistical analysis

Student two-tailed *t* test was used to evaluate significance of two groups. GraphPad Prism (GraphPad, La Jolla CA, USA) was used to determine statistical significance and generate graphs and plots. A *p value* < *0.05* was considered statistically significant; * is *p* < *0.05*, ** is *p* < *0.01* and *** is *p* < *0.001*. ns = not significant. Error bars for all figures indicate standard errors. Survival data was plotted using the Kaplan–Meier method. Significance of differences between groups were tested by comparing group means and medians (mean survival time [MST]) by either the two-tailed *t* test or Wilcoxon’s signed-rank test.

## Results

### Infiltration of autoreactive T cells in implanted islet-seeded biomaterial within NOD diabetic recipient mice

**Fig. 1:**
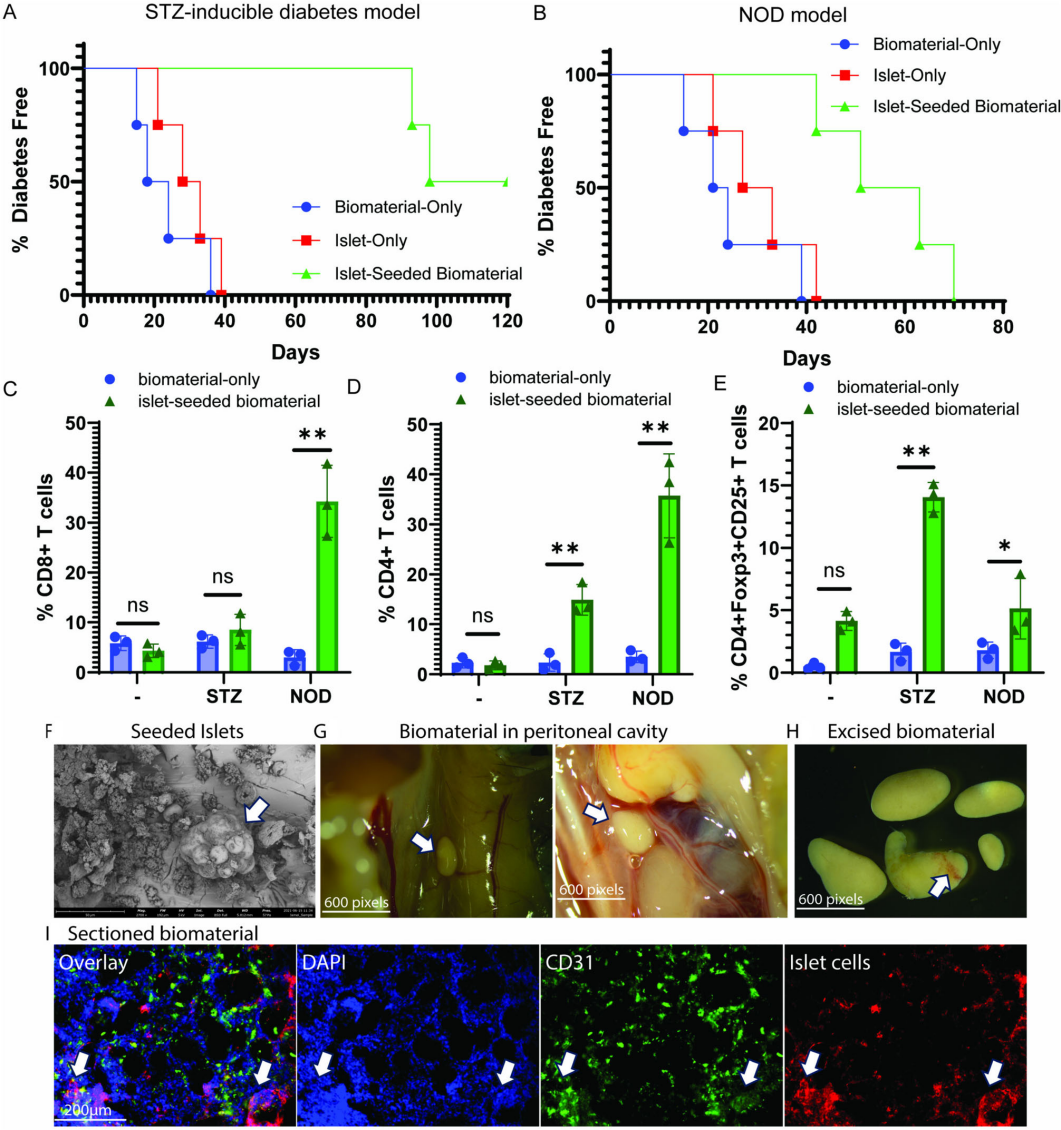
Islet-seeded biomaterial in NOD diabetic recipients contains high proportion of effector T cells and low levels of Tregs. Streptozotocin-induced or NOD pre-diabetic mice received either intraperitoneal implantation of biomaterial-only, islets-only or islet-seeded biomaterial. B6 or NOD.SCID mice served as (-) controls. The time of initial implantation represents time (day) 0. **A**, **B** Glucose was measured every 3 days for the first 45 days and then every 7 days through the end of the experiment period. Animals were diabetes free if two consecutive readings resulted in blood glucose levels below 300 mg/dL. **C**–**E** STZ-induced or NOD pre-diabetic mice implanted with biomaterial-only or islet-seeded biomaterial were sacrificed 30 days post-implantation. Biomaterial was dissociated into single cell suspension prior to analyses by flow cytometry. Samples were gated on MHC class I + subsets and assessed for CD8 + T cells, CD4 + T cells and the percentage of CD4 + CD25 + Foxp3 + T cells. All gates were established using negative and single color positive controls. Biomaterial seeded with islets was imaged via **F** electron microscopy analysis pre-implantation into mice. Next, biomaterial was dissected from treated mice 8 weeks post-implantation and identified using **G**, **H** stereomicroscope visualization; arrows indicate location of biomaterial pieces and identified vasculature around biomaterial piece. **I** Excised biomaterial pieces were cryosection and stained for fluorescence microscopy analysis of DAPI, CD31 and Far Red labeled islets. Arrows indicate remained islets embedded within the excised biomaterial

Prior published reports by our research group demonstrated success in use of a novel microcarrier seeded with syngeneic islets to reverse diabetes in mice [8]. Using both the streptozotocin (STZ)-inducible and the genetically-predisposed diabetic mice, prior successful implantation of islets-seeded in the biomaterial within hyperglycemic mice formed a stable organoid structure within the peritoneal cavity [8]. Within the STZ mouse model, over 50% of mice remained diabetes free for 120 days post-implantation of the islet-seeded biomaterial (Fig. 1A). Control groups containing biomaterial-only or islets-only injected i.p. all succumbed to diabetes, as measured by two consecutive weeks above 300 mg/dL, within 40 days. However, the NOD diabetic recipient of islet-seeded biomaterial was only able to stave off being diabetes free for about 70 days (Fig. 1B). To further delineate the cause of the islet-seeded biomaterial failure to long-term restrain diabetes onset in the NOD model, the biomaterial was resected at 30 days post-implantation in the biomaterial-only and islet-seeded biomaterial recipient groups. Assessment of infiltrated T cells revealed a much higher proportion of CD8 + and CD4 + T cells in the NOD recipients compared to STZ treated cohorts (Fig. 1C and D). STZ-induced diabetic mice receiving islet-seeded biomaterial had an average of 8.5% ± 1.9 CD8 + T cells, whereas NOD mice recipients had a mean of 34.2% ± 4.3 30 days after implantation of islet-seeded biomaterial. Non-diabetic B6 and NOD.SCID mice served as internal controls. Similarly, STZ-induced diabetic recipients had a mean of 14.8% ± 2 CD8 + T cells infiltrated into the resected islet-seeded biomaterial compared to NOD recipients having a mean of 35.7 ± 4.8. This collectively showed greater infiltration of T cells into the transplanted islet-seeded biomaterial. Interestingly, further analyses of the CD4 + T cell populations revealed that the STZ-inducible diabetic recipient of the islet-seeded biomaterial had threefold higher frequency of CD4 + Foxp3 + Tregs compared to the NOD groups (Fig. 1E). Furthermore, results from this work showed recapitulated formation of an islet-biomaterial aggregation prior to implantation (Fig. 1F) that eventually formed into an organoid-like structure with neovascularization in the excised islet-seeded biomaterial from NOD treated mice (Fig. 1G–I). Collectively, these analyses suggested that the inherent autoimmune nature of the NOD diabetic mouse resulted in aggressive infiltrative of autoreactive T cells into the implanted islet-seeded biomaterial.

### Implant of islet + Treg co-seeded biomaterial into diabetic mice restores normoglycemia

**Fig. 2:**
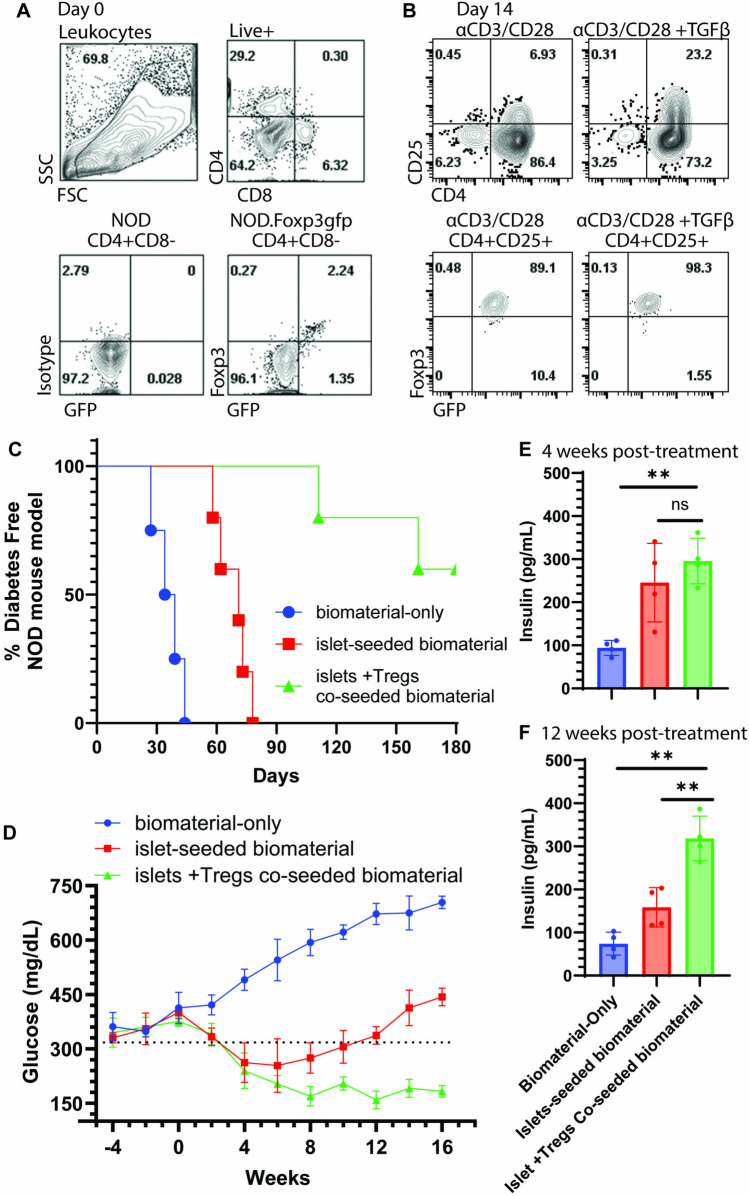
Co-seeding of islets and Tregs into the biomaterial sustains normoglycemia. **A**, **B** NOD-Foxp3^gfp^ mice bearing the GFP reporter under the Foxp3 promoter, were used to expand and FACS-sort CD4^+^CD25^+^Foxp3gfp^+^ Tregs. To confirm immunosuppressive functionality, expanded Foxp3^gfp+^ subsets were cultured with NOD pancreas-derived beta cells and pancreas-derived effector CD8 + T cells *in vitro *. **C** NOD pre-diabetic mice received either biomaterial-only, islet-seeded biomaterial or biomaterial co-seeded with islets and Treg (islet + Tregs co-seeded biomaterial). Time of implantation represents time (day) 0. Non-fasting glucose levels were measured through the course of the experiment. Two consecutive glucose readings below 300 mg/dL identified mice as being diabetes free; readings above 300 mg/dL were considered positive for diabetes. **D** Diabetic NOD mice that registered approximately 400 ± 31 mg/dL of non-fasting blood glucose were implanted with either biomaterial-only, islet-seeded biomaterial or islets and Tregs co-seeded in the biomaterial. Each cohort had 3 NOD diabetic recipients. Non-fasting blood glucose levels were then measured every two weeks 16 weeks post-implantation. (**E**, **F**) Insulin levels were measured at 4 and 12 weeks post-implantation with the biomaterial-only, islet-seeded biomaterial and islet + Tregs co-seeded biomaterial by Luminex multiplex analyses and corroborated with standard ELISA assays

Previous works have shown that pancreas-resident Tregs were important for long-term islet grafts in models of T1D [37–39]. Observing that endogenous circulating autoreactive CD8 + and CD4 + T cells infiltrate into the islet-seeded biomaterial, studies next set to employ Tregs as countermeasures to foster an immune tolerance microenvironment within the organoid. NOD-Foxp3^gfp^ mice, which bear the GFP reporter under the Foxp3 promoter, were used to expand and FACS-sort CD4^+^CD25^+^Foxp3gfp^+^ Tregs following approaches described by Chen et al. [7] (Fig. 2A, B). To confirm immunosuppressive functionality, expanded Foxp3^gfp+^ subsets were cultured with NOD pancreas-derived beta cells and pancreas-derived effector CD8 + T cells i*in vitro*. In presence of the Tregs, the effector CD8 + T cells had less *in vitro* killing potential, reduced IFNγ production and lower levels of Granzyme-B.

The biomaterial was prepared by *in vitro* co-seeding of syngeneic beta cells and Foxp3^gfp^. The cell-seeded biomaterial was then intraperitoneal injected into pre-diabetic recipient NOD mice. Methods of implantation are previously described and demonstrated in prior published works [8]. Pre-diabetic mice were defined as transient readings between 100 and 150 mg/dL non-fasting glucose levels. Diabetic mice were defined as two consecutive weeks of 300 mg/dL or greater [34,35]. Studies then monitored the ability of the implanted biomaterial co-seeded with Tregs and islets to restrain diabetes onset compared to islet-seeded biomaterial (without Tregs) and biomaterial-only groups. Results revealed that introduction of Tregs along with islets in the biomaterial resulted in ~ 60% of mice being free of diabetes for greater than 180 days after implantation treatment (Fig. 2C). The remaining 40% that did succumb to diabetes had an average onset of 150 days post-treatment, with the islet-seeded biomaterial having an average onset of 73 days and the biomaterial-only group 34 days. All mice within the islet-seeded biomaterial and biomaterial-only groups developed diabetes. Next, to evaluate the ability to reverse diabetes onset, diabetic NOD mice at approximately 400 mg/dL were implanted at week 0 with islets and Tregs co-seeded in the biomaterial. Compared to biomaterial-only and islet-seeded biomaterial groups, the islet + Treg co-seeded biomaterial reduced hyperglycemic levels to an average of 165 mg/dL of non-fasting glucose levels for 16 weeks post-implantation (Fig. 2D). Although the islet-seeded biomaterial did reverse diabetes for approximately 8 weeks, the implant failed for long-term sustained normal glycemia with levels increasing above 300 mg/dL at 10 weeks post-implantation. Serum measurements of non-fasting insulin levels at 4 weeks post-treatment revealed elevated levels of 245 ± 50.25 and 295.8 ± 52.78 pg/mL in both the islet-seeded and islet + Treg co-seeded biomaterial compared to 94.1 ± 46.47 pg/mL in the biomaterial-only control (Fig. 2E). Levels at 12 weeks post-implantation revealed a drop of non-fasting insulin levels in the islet-seeded biomaterial group to 158.5 ± 34.5, whereas the islet + Treg co-seeded biomaterial recipients had non-fasting insulin level at 318.3 ± 160 pg/mL (Fig. 2F).

### Restored metabolic indices in diabetes mice receiving islet-Treg co-seeded biomaterial

**Fig. 3:**
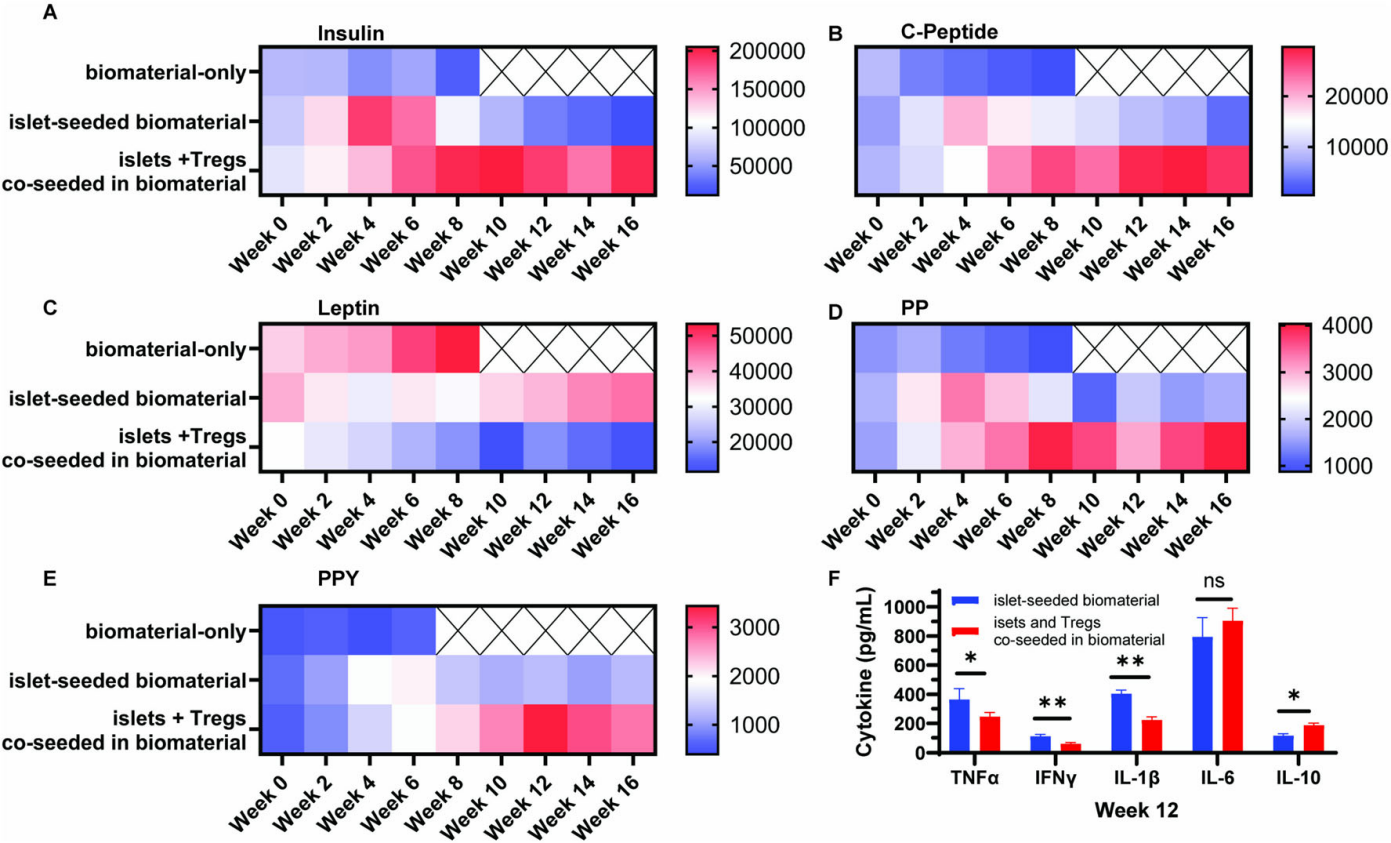
Normalization of metabolic levels and reduction of pro-inflammatory signature in NOD diabetic recipients receiving islet + Treg co-seeded biomaterial. Diabetic NOD mice that registered approximately 400 ± 31 mg/dL of non-fasting blood glucose were implanted with either biomaterial-only, islet-seeded biomaterial or islets and Tregs co-seeded in the biomaterial. Blood serum was collected from recipients every two weeks post-implantation. Metabolic panels for **A** insulin, **B** c-peptide, **C** leptin, **D** pancreatic polypeptide (PP) and **E** peptide YY (PYY). Results are presented in a heat map measuring levels over the course of 16 weeks post-implantation. There were three mice for each cohort. F Serum cytokine levels at week 12 were assessed within islet-seeded biomaterial and the islet + Treg coseeded biomaterial groups for TNFα IFNγ, IL-1β, IL-6 and IL-10. Studies evaluated the serum of three mice of each cohort in triplicate

Diabetic NOD recipient mice that received either the islet-biomaterial or islet + Treg co-seeded biomaterial had normalized metabolic indices immediately post-implant, as measured by levels of insulin, C-peptide, Leptin, PP, and PPY (Fig. 3A–E). However, the islet-biomaterial group deviated from normalization approximately 6 weeks after implantation and reverse course back to deregulated levels. This mirrors previous findings upon i.p. Injection of islets co-seeded in the biomaterial [8], In turn, NOD recipients of the islet + Treg co-seeded biomaterial had sustained normalization of metabolic indicators through week 16 of the study. Additional measurement of serum cytokine levels revealed depressed TNFα, IFNγ and IL1β in the islet + Treg co-seeded biomaterial cohort at week 12 post-implantation compared to islet-biomaterial only diabetic recipients (Fig. 3F). There was also an observed increase in IL-10 serum levels in the islet + Treg co-seeded biomaterial cohort compared to control groups. No significant changes were observed in IL-6.

### Suppression of autoimmune assault on the implanted islet-Treg-seeded biomaterial

**Fig. 4:**
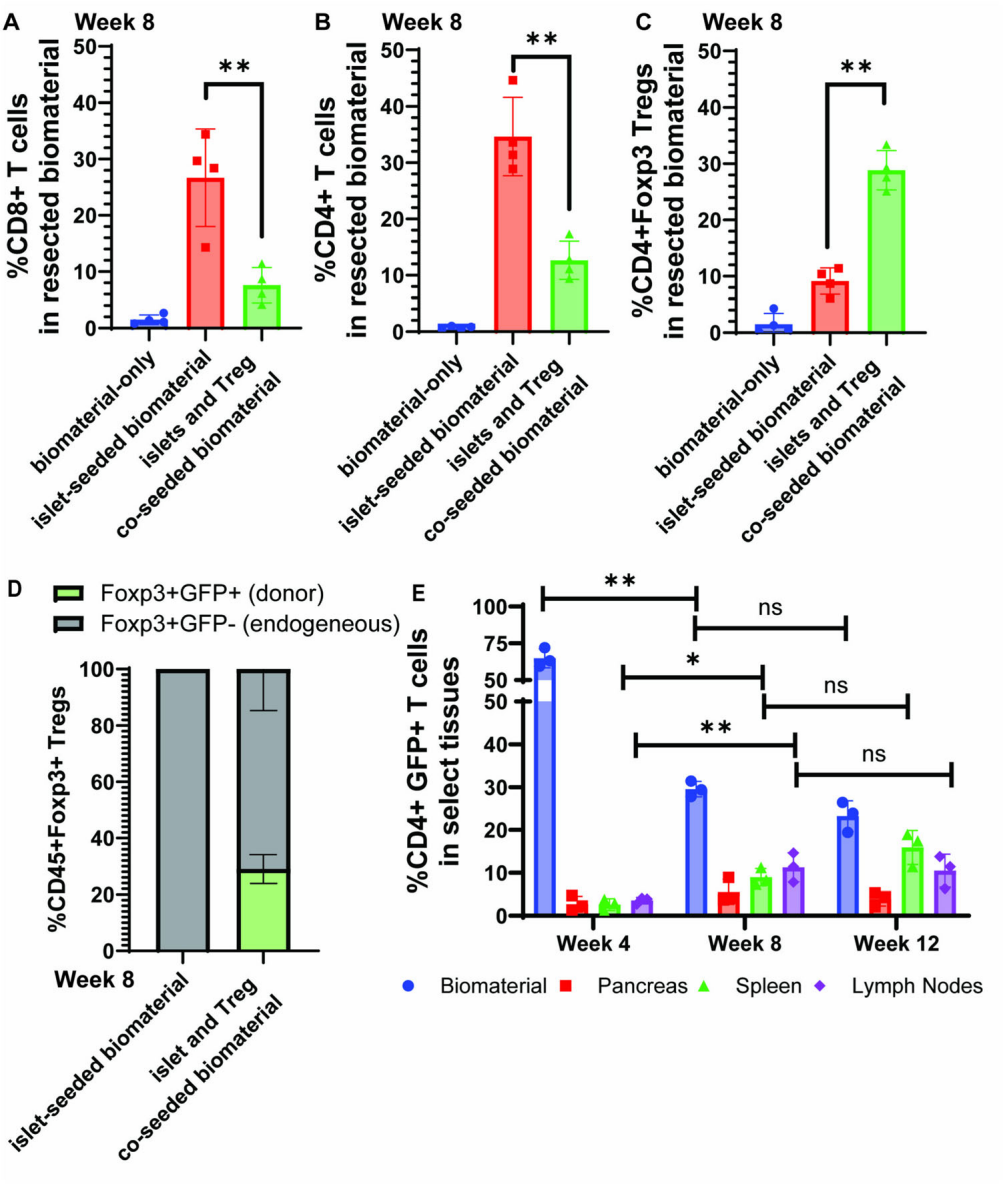
*In vivo* implantation of Tregs and islets seeded in the biomaterial effectively reduces hyperglycemia and limits autoimmune pathology in NOD mice. NOD diabetic mice receiving the implantation of biomaterial-only, islet-seeded biomaterial or islet + Treg co-seeded biomaterial were sacrificed after 8 weeks post-implantation. The biomaterial organoid was then dissociated to prepare single cell suspension of total cells. **A**, **B** Using flow cytometric analyses, the percentage of CD8 + and CD4 + T cells infiltrating into the biomaterial was assessed. **C** The percentage of Foxp3 + subsets within CD4 + T cells was further assessed. To assess the percentage of endogenous Foxp3 + Tregs compared to those initial donor Tregs co-seeded into the biomaterial, studies initially seeded Tregs derived from the NOD-Foxp3^gfp^ mouse bearing the GFP reporter under the Foxp3 promoter (Foxp3^gfp^ + Tregs) for implantation into conventional NOD diabetic recipients. **D** At week 8 post-implantation, flow cytometric analysis was performed to determine the proportion of Foxp^gfp^ Tregs, which served as donors by *in vitro* seeding into the biomaterial prior to implantation, and the Foxp3 + Tregs absent of GFP expression, which represent the recipient endogeneous Treg populations, within the islet-seeded biomaterial group compared to the islet + Treg co-seeded biomaterial recipient cohort. E Pancreas, spleen and lymph node tissues, in addition to the biomaterial implant, was harvested at weeks 4, 8 and 12 post-implantation to assess the efflux of the Foxp3^gfp^ + Tregs out of the initially seeded biomaterial and in to other tissues. Three mice were assessed within each cohort

Resected implants from diabetic recipient mice receiving islet + Treg co-seeded biomaterial revealed depressed levels of infiltrating CD4 + and CD8 + T cells compared to recipients receiving the islet-biomaterial. At 8 weeks post-implantation into recipient diabetic mice, resected islet-biomaterial had 26.7% CD8 + T cells, whereas recipients receiving islet + Treg co-seeded biomaterial had 7.6% (Fig. 4A). Similarly, islet-biomaterial had 34.6% CD4 + T cells compared to the reduced frequency of 12.6% in the islet + Treg co-seeded biomaterial (Fig. 4B). Further analyses of the total CD4 + T cell population revealed that 28.8% was CD4 + Foxp3 + Tregs in the islet + Treg co-seeded biomaterial compared to only 9.1% in the islet-biomaterial group (Fig. 4C). Interestingly, a greater proportion of these CD4 + Foxp3 + Tregs within the islet + Treg co-seeded biomaterial were endogenous of the recipient, with 71.4% of Tregs infiltrated from other compartments (Fig. 4D). 28.6% of Tregs were Foxp3^gfp^ as assessed on week 8 post-implantation. As expected, no Foxp3^gfp^ + cells were detected in the islet-seeded biomaterial group. Lastly, to assess the efflux of the seeded Foxp3^gfp^ + Tregs out of the islet + Treg co-seeded biomaterial post-implantation, the pancreas, spleen and lymph node tissues were collected at weeks 4, 8 and 12 and assessed for the proportion of Foxp3^gfp^ + subsets within gated CD4 + subsets. Results revealed rapid decrease in the total frequency of seeded Foxp3^gfp^ + Tregs from total pool of CD4 + Foxp3 + Tregs in the biomaterial from 64.8% at week 4, to 29.4% in week 8 and 23.3% by week 12 (Fig. 4E). Inversely, there was a concomitant increase in the amount of the Foxp3^gfp^ + Tregs that have exited out of the islet-Treg co-seeded biomaterial and entered into the spleen and lymph nodes. For the spleen, the frequency of Foxp3^gfp^ + Tregs among total Tregs increased from 2.6% in week 4, to 8.9% in week 8 and 15.9% by week 12. Similarly, the frequency of Foxp3^gfp^ + Tregs increased in the lymph nodes from 3.5% at week 4 to 11.27% by week 8 and 10.5% in week 12. No significant numbers of Foxp3^gfp^ + Tregs were found to infiltrate into the pancreas.

### Islet + Treg co-seeding in biomaterial prevents diabetes, but not injection of free Tregs

**Fig. 5:**
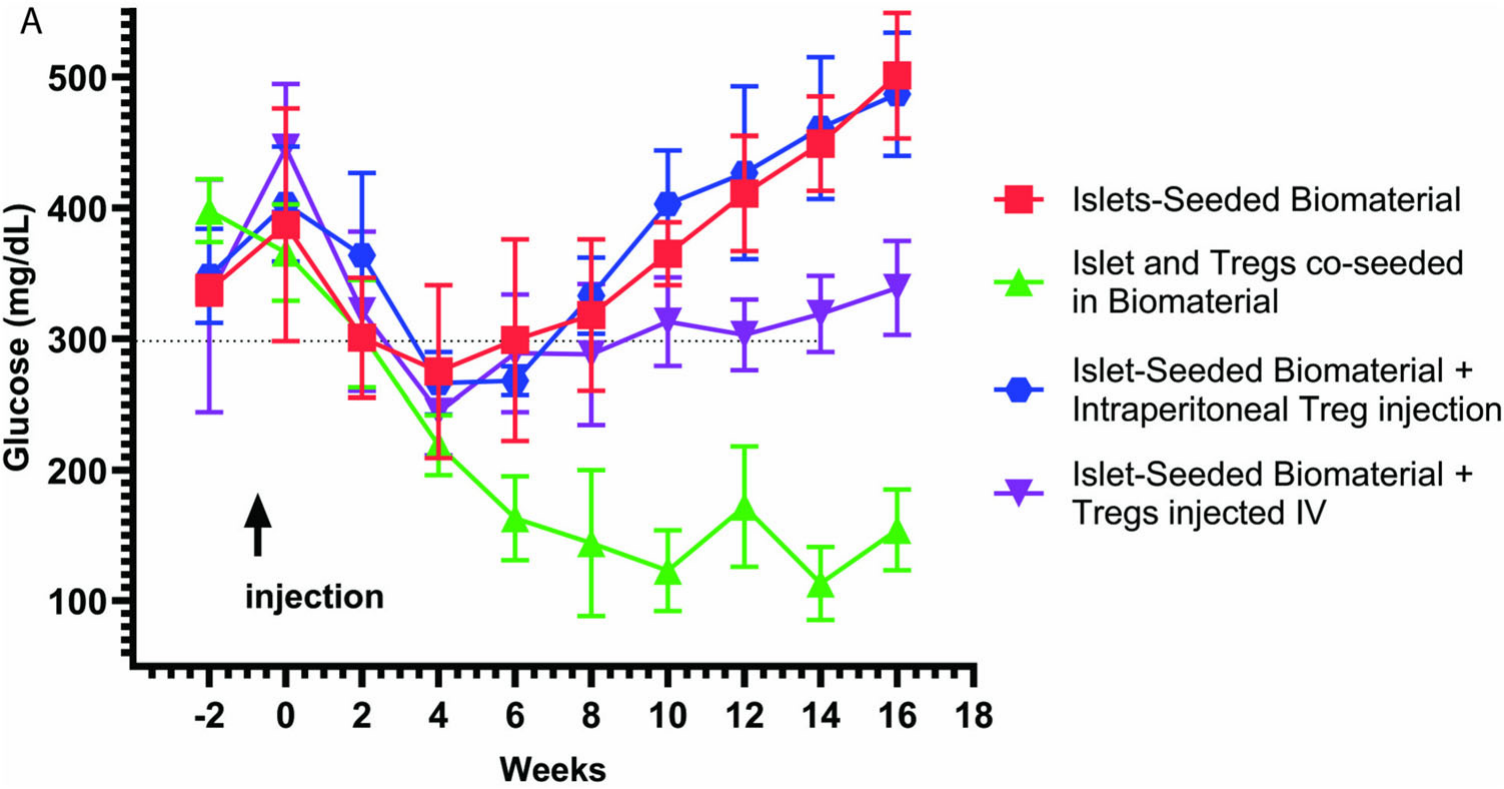
Exogenous addition of islets in tandem with implanting islet-seeded biomateria is not effective in reversing diabetes compared to co-seeding of islets anssd Tregs directly into the biomaterial for implantation. NOD diabetic mice were implanted with islet-seeded biomaterial or islets and Tregs co-seeded in biomaterial. Additional groups received islet-seeded biomaterial i.p. Implanted in conjunction with Tregs injected intraperitoneally and a final group of islet-seeded biomaterial i.p. implanted in tandem with Tregs intravenously injected. Non-fasting glucose measurements were taken every two weeks through the 18 week experiment. Each cohort had two mice and the study was repeated twice.

Given that the islet + Treg co-seeded biomaterial had reduced frequency of the Foxp3^gfp^ + Tregs after successive weeks post-implantation in diabetic recipients, studies next assessed if exogenous addition of Tregs in conjunction with implanting islet-seeded biomaterial could yield the same effect as islet + Tregs co-seeded in the biomaterial. To directly address, diabetic recipient mice cohorts received implantation of islet-seeded biomaterial in tandem with intravenous or intraperitoneal injection of the Foxp3^gfp^ + Tregs (Fig. 5). Islet-seeded biomaterial in tandem with intraperitoneal Treg injection had no impact differential than recipients receiving islet-seeded biomaterial alone. Notably, the intravenous injection in tandem with implanting islet-seeded biomaterial was able to moderately restrain diabetes progression. However, the co-seeding of islets and Tregs was able to adequately reverse elevated glucose levels through the 16-week observed period post-treatment.

## Discussion

Diabetic NOD mice are plagued with chronic autoimmune infiltration of effector T cells that specifically target destruction of the pancreatic beta cells. Prior works have established that intraperitoneal implantation of a biomaterial seeded with islets was able to restore normoglycemia in both the STZ-inducible and NOD diabetic mice models [8]. Although long-term normoglycemia was sustained post-implantation with the islet-seeded biomaterial in STZ-inducible diabetic mice, NOD diabetic recipients only were able to restore normal glucose levels for several weeks post-implantation. As occurs in the clinical setting, long-term outcomes of islet transplantation were met with rejection due to allo- and/or autoimmune-mediated responses [10]. Studies further revealed that autoreactive T cells infiltrated the implanted islet-seeded organoid, resulting in destruction of the compartment and failure to produce sufficient insulin in response to rising glucose levels. Thus, establishing immune tolerance within the islet-seeded organoid compartment was required to restrain infiltration and/or inhibit autoreactive T cell destruction of the beta cells.

Several studies have shown the importance of CD4 + CD25 + Foxp3 + Tregs in restraining autoimmunity in T1D [40–44]. Whether in absence or defect in immunoregulatory functions, Tregs have been demonstrated to be critical in restraining autoreactive T cell destruction of the beta cells in the pancreas compartment. However, Tonkine DR et al., [45] have shown that transfer of polyclonal Tregs is partially (but not fully) able to restrain effector responses. Thus, the approach is variable and only effective at a high Treg to autoreactive T cell ratio. The number of Tregs to efficiently restrain immunity is directly dependent on the number of autoreactive T cells circulating. Thus, any infiltrating autoreactive T cell entering into the islet + Treg co-seeded biomaterial will be met by high numbers of the Tregs that will effectively inhibit their autoimmune responses.

To evaluate this phenomena, Tregs were *in vitro* expanded from lymph node-derived naive T cells prior to co-seeding in the biomaterial along with syngeneic islets. The approach generated a polyclonal population of Tregs. Notably, studies have shown that sufficiently large populations of polyclonal Tregs does contain enough diverse antigen-specific Tregs directed to the beta cell (and pancreas compartment) to suppress diabetes [45]. Results generated herein corroborate success in use of polyclonal Tregs within the biomaterial, with implantation protecting the viability of the implanted beta cells to continually produce insulin in response to glucose for 30 weeks post-treatment.

Co-transplantation of Tregs within pancreatic islet transplants has been proposed to be an efficient means to protect graft tissue survival [46]. Prior studies using a PLG scaffold system have shown successful delivery of antigen-specific Tregs to limit tissue pathology in islet grafts [47]. Other studies have attempted to incorporate anti-inflammatory agents within the encapsulated biomaterials seeded with islets cells [48]. Notably, clever approaches have engineered encapsulated biomaterials or gene-edited beta cells to carry/produce chemokines (i.e. CCL12) for recruitment of the systemically injected antigen-specific Tregs [15,49,50]. To directly address the challenges regarding autoreactive T cell infiltration into transplanted islets, the studies herein co-seeded syngeneic islets in a novel biomaterial along with Foxp3 + Tregs for adoptive i.p. implantation in diabetic mice. The value of this biomaterial is that it can embed cells to establish a stable microenvironment and provide physical protection for transplanted cells. The biomaterial possesses a solid surface and low density that allows both external and internal cells to move freely through the interspace of the fibrous micro biomaterial. Implantation of co-seeding of the islets and Tregs within the biomaterial was able to sufficiently reduce glucose levels in hyperglycemic NOD recipients. Notably, islet-only seeded biomaterial was also able to restore normoglycemia for a few weeks post implantation. However, this reproducibly failed after 6–8 weeks post-implantation, as evidenced by the sharp rise of glucose and depressed levels of non-fasting insulin levels. In turn, co-seeding of the islets and Tregs in the biomaterial allowed the organoid to normalize glucose levels and sustain high non-fasting insulin levels through the entire 16-week post-implantation study. Thus, results demonstrates that initially co-seed the islets with the Tregs in the implanted biomaterial results in an effective approach to inhibit the autoreactive T cells infiltrating into the biomaterial. This successfully resulted in achievement of normoglycemia in tandem with restored healthy metabolic levels, as measured by restored C-Peptide, Leptin, PP and PPY levels, through the length of the study.

In tandem, studies observed a reduction in several pro-inflammatory cytokines, such as TNFα, IFNγ and IL-1β, in NOD mice that received the islets and Treg co-seeded biomaterial compared to recipients that received only islets seeded in the biomaterial. This reinforces the importance of Tregs in establishing tolerance within the compartment. As previously published, implantation of the cell-seeded biomaterial in diabetic recipients resulted in rapid neovascularization and formation of an organoid structure [8]. Although formation of blood vasculature is crucial for monitoring glucose and responsive secretion of insulin into the blood, these same vessels also serve as principal routes for leukocyte trafficking. However, inclusion of the Tregs into the biomaterial with the islets would protect them from the influx of pro-inflammatory myeloid cells, autoreactive T cells and the breadth of cytokine and chemokine factors in the blood. This would further shift the environment from a pro- to an anti-inflammatory state, as measured by reduced pro-inflammatory molecules coupled with increase in IL-10 within the serum of recipients receiving the islet and Treg co-seeded biomaterial. Notably, IL-6 levels were not altered among the cohorts implanted with either islet-seeded vs. the islet + Treg co-seeded biomaterial. This was not surprising given reports that have shown that IL-6 activities have limited contribution to development of T1D [51–53].

The exit of such a large portion of the seeded Foxp3^gfp^ + Tregs post-implantation in diabetic recipients may suggest that only antigen-specific subsets are retained, leaving the majority of the expanded polyclonal Treg subsets exiting into the periphery and populating other organs. In concert, creation of this initial immunosuppressive environment may have supported the influx and expansion of endogenous Tregs to further foster the immunosuppressive environment within the islet-seeded organoid. This endogenous recruitment may additionally offset the functional instability of *in vitro* generated Tregs, which may have led to their depressed numbers or destabilization of sustained Foxp3 expression. Thus, methods to induce islet-specific Tregs *in vitro* that are sufficiently driven through the T cell receptor (TCR) may further enhance long-term retention within the biomaterial upon implantation and help to recruit other endogenous Tregs to reinforce tolerance within the implanted organoid. However, for clinical-relevant therapies, novel methods to enrich the small pool of islet-specific Tregs or convert T effector cells into these suppressive cells remains a challenge.

Taken together, these studies highlight the importance of developing clinical-relevant therapies for restoring normoglycemia in T1D. Use of a novel organoid system composed of donor islets and ex vivo expanded Tregs presents a viable approach to efficiently reverse diabetes. Importantly, the presence of Tregs are paramount to prevent circulating autoreactive T cells, initially responsible for destroying the beta cells within the pancreas compartment, to infiltrate the implanted islet-seeded organoid. Notably, co-intravenous or -intraperitoneal delivery of Tregs in tandem with implanting the islet-seeded biomaterial were inefficient in long-term rescue of the graft; co-seeding of the islets and Tregs was required for long-term viability of restoring normoglycemia post-implantation. This may simply be due to the necessity to create an local immunosuppressive microenvironment within the biomaterial housing the beta cells. Whereas intraperitoneal or intravenous injection dispersed the Tregs throughout the body leaving an insufficient amount localized within the biomaterial to protect against the infiltrating autoreactive T cells. Thereby, this approach indirectly reinforces much of the field aim to create encapsulated biomaterials containing immunosuppressive agents in conjunction with insulin-producing cells to curb autoimmune events.

## Data Availability

The datasets during and/or analyzed during the current study available from the corresponding author on reasonable request.
